# Anti-inflammatory drugs suppress ultrasound-mediated mesenchymal stromal cell tropism to kidneys

**DOI:** 10.1038/s41598-017-08887-x

**Published:** 2017-08-17

**Authors:** Scott R. Burks, Ben A. Nguyen, Michele N. Bresler, Matthew E. Nagle, Saejeong J. Kim, Joseph A. Frank

**Affiliations:** 1 Frank Laboratory, Department of Radiology and Imaging Sciences, National Institutes of Health Clinical Center, Bethesda, MD 20892 USA; 2 National Institute of Biomedical Imaging and Bioengineering, Bethesda, MD 20892 USA

## Abstract

Mesenchymal stromal cells (MSC) are potential renal therapeutics. Clinically, results are mixed partly because MSC tropism to kidneys is minimal following infusion. Ultrasound augmentation of the renal microenvironment is becoming increasingly-important in renal MSC therapies. We demonstrated pulsed-focused-ultrasound (pFUS) increases enhanced homing permeability and retention of MSC in mouse kidneys. Here, we characterized the temporal proteomic response to pFUS in mouse kidneys and its association with MSC tropism. pFUS induced molecular cascades of initial increases in tumor necrosis factor-α (TNFα) and interleukin (IL)-1α, that activated nuclear factor kappa-light-chain-enhancer of activated B cells (NFκB) and cyclooxygenase-2 (COX2) pathways without cell death. This was followed by a 24–48 hour-long response of increased cell adhesion molecules (CAM), trophic and anti-inflammatory factors. Pretreating animals with anti-inflammatory drugs etanercept (TNFα inhibitor), anakinra (IL-1 receptor antagonist), prednisone (NFκB translocation inhibitor), or ibuprofen (COX inhibitor) suppressed molecular changes and inhibited renal MSC tropism. We further examined the role of COX2 using a COX2-knock-out mouse where pFUS was unable to increase MSC tropism. These results demonstrate that renal micro-environmental changes induce MSC tropism and could influence the therapeutic efficacy of MSC. Optimizing the microenvironment and understanding drug effects will enable improvements in MSC therapies for renal disease.

## Introduction

Mesenchymal stromal cells (MSC), also known as mesenchymal stem cells, have been used to treat a variety of renal pathologies. Preliminary clinical data have shown promising effects of MSC, but remain inconclusive regarding their effectiveness in renal diseases^1^. Preclinical studies have elucidated many therapeutic mechanisms that typically involve immunomodulatory and paracrine effects from transplanted MSC^2^. Moreover, some mechanisms of action require tropism of MSC to the kidney for effective modulation of the inflammatory microenvironment^3^. Renal cytokines, chemokines, trophic factors (CCTF), and cell adhesion molecules (CAM) work in concert to promote extravasation of MSC through mechanisms that are thought to be analogous to leukocyte homing^4^. While many factors can influence MSC therapies, local microenvironmental changes may play a role in MSC tropism to kidneys and influence therapeutic function.

We have investigated the molecular effects of pulsed focused ultrasound (pFUS) in different tissues and demonstrated that through mechanotransduction, sonication alters the microenvironment of targeted tissues^5–11^, enhances tropism of MSC^5, 7, 9–11^, and acts as a neo-adjuvant in acute kidney injury (AKI)^5^ and limb ischemia models^10^. We have previously demonstrated that sonicating mouse kidneys with pFUS stimulated enhanced homing permeability and retention (EHPR) of intravenously-administered MSC without adverse effects^11^. pFUS combined with MSC infusions were used in a mouse model of cisplatin-induced acute kidney injury to enhance renal protection, survival, and recovery of renal function. Moreover, the therapeutic potency of MSC depended on upregulated cytokines in the renal microenvironment to condition cells after they migrated to kidneys^5, 10^.

The renal microenvironment is critical to MSC tropism and improved potency, and can be modulated by ultrasound, which is playing an ever-increasing role in renal regenerative medicine. Therefore, it is vital to characterize renal parenchymal changes following pFUS. Furthermore, we sought to use clinically approved drugs as selective pharmacological inhibitors to both understand the necessary molecular cues that induce pro-homing signaling, but also demonstrate the effects that routinely-employed drugs might have on conditions and their potential impacts on MSC therapies.

Here, we describe the temporal changes in CCTF and CAM following pFUS to mouse kidneys. We demonstrate a molecular cascade in renal tissue that is characterized by early elevations in tumor necrosis factor-α (TNFα), interleukin (IL)-1α, followed by later upregulation of nuclear factor kappa-light-chain-enhancer of activated B cells (NFκB) and cyclooxygenase-2 (COX2) pathways. Since the initial pro-inflammatory molecular responses are necessary to induce an EHPR of MSC following pFUS, it was important to understand the influence of anti-inflammatory drugs on the renal microenvironment and their potential impact on MSC tropism to targeted sites. When mice were pretreated with drugs to inhibit nuclear translocation of NFκB (prednisone), or inhibit TNFα (etanercept), IL-1α (anakinra), or COX (ibuprofen), we demonstrated that the pFUS-induced molecular cascade was suppressed and fewer MSC homed to sonicated kidneys. In COX2 knockout (KO) mice, pFUS followed by MSC infusion resulted in decreased numbers of MSC homing to kidneys compared to wild-type control mice. These data illuminate how different therapeutic approaches in renal regenerative medicine may conflict with each other and provide some insight into the heterogeneity of clinical MSC data. Understanding interactions between different therapeutic tools will be critical to develop effective clinical MSC therapies of renal diseases.

## Results

### Proteomic response to pFUS in normal kidney

**Figure 1:**
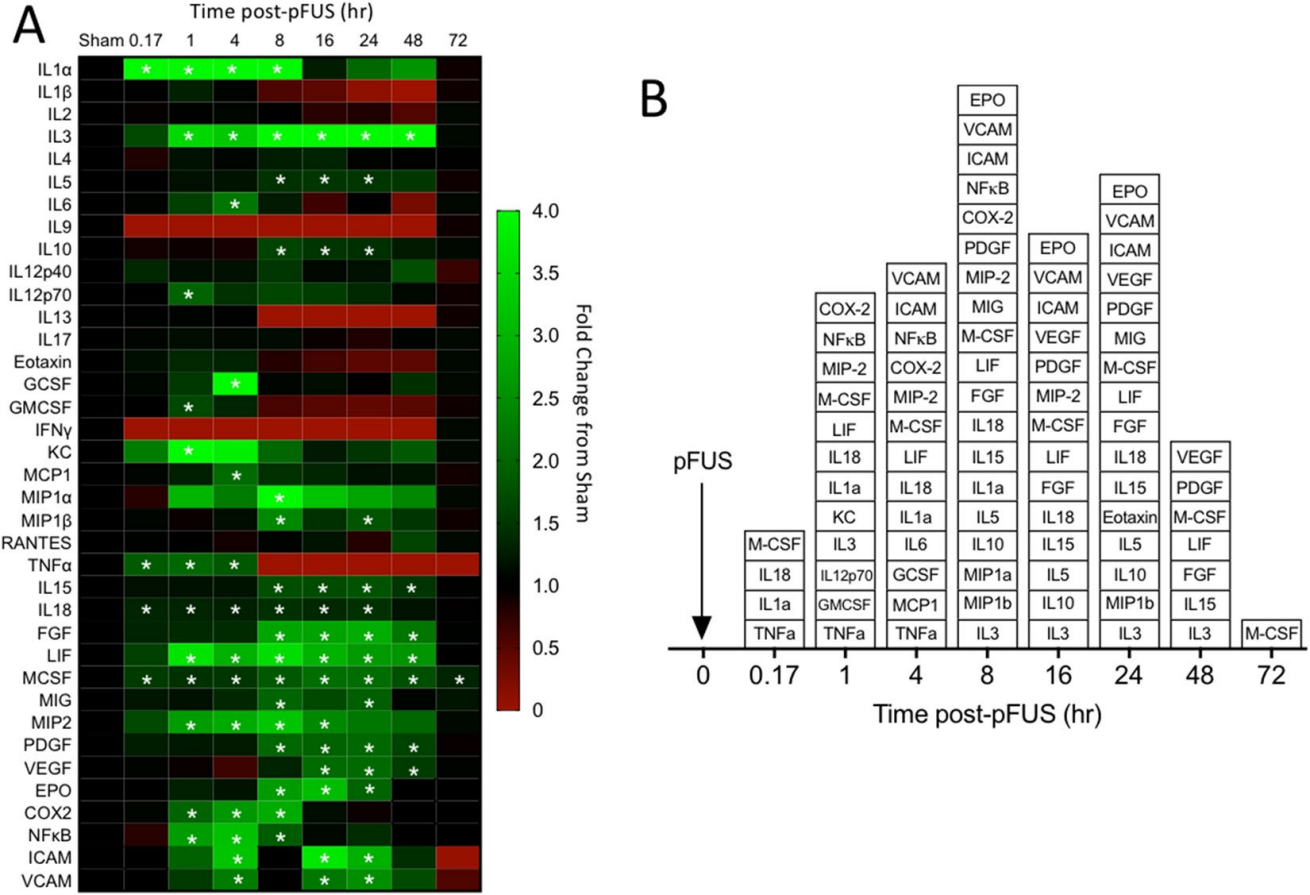
Heat map and box plot of molecular response to pFUS in the kidney. (**A**) Heat map shows fold changes at each time point compared to sham controls (no pFUS). Increases are indicated in green while decreases are shown in red. White asterisks indicate elevations that are statistically significant compared to sham controls (p < 0.05 by ANOVA with Bonferroni post-hoc tests). (**B**) Box plot of significant increases at each time point.

Mice were administered pFUS to a single kidney and then euthanized at 10 min, 1, 4, 8, 16, 24, 48, and 72 hours post-pFUS (n = 6 mice per time point). Sham-treated control mice (n = 6) underwent the pFUS protocol, but the transducer remained off during the sonication portion of the protocol. pFUS elicited a complex molecular response from 10 min through 72 hr post-sonication (heat map and graphical representation shown in Fig. 1; see Supplement for raw data). The molecular response is defined by early upregulation of pro-inflammatory cytokines that subsided and then gave way to expression of more anti-inflammatory cytokines, chemokines and trophic factors. Statistically significant elevations were determined using an ANOVA with Bonferroni corrections (p < 0.05) compared to sham control kidneys. Notably, pFUS transiently elevated TNFα, IL-1α, and IL-18 along with macrophage colony stimulating factor (M-CSF) at 10 min. Increased TNFα expression persisted for 4 hours, while M-CSF remained elevated at all time points through 72 hr post-pFUS. At 1 hr post-pFUS significant elevations in IL 3, IL-12, COX2, NFκB, and keratinocyte chemoattractant (KC) macrophage inflammatory protein (MIP), and granulocyte macrophage CSF (GM-CSF) were detected for up to or more than 8 hours. At 4 hr post pFUS, monocyte chemoattractant protein-1 (MCP-1) and IL-5 became elevated, as well as intercellular adhesion molecule (ICAM) and vascular cell adhesion protein (VCAM). A transition in the molecular profile occured at 8 hr post-sonication. Pro-inflammatory markers such as TNFα, IL-1α, IL-12, KC, and NFκB were no longer elevated, and anti-inflammatory cytokines and growth factors became significantly increased like IL-10, IL-15, fibroblast growth factor (FGF), platelet-derived growth factor (PDGF), and erythropoietin (EPO) starting at 8 hr post-sonication. By 16 hr post-pFUS, IL-1α and COX2 were not elevated, and vascular endothelial growth factor (VEGF) was. At 24 hr post-pFUS, the profile consisted almost entirely of anti-inflammatory CCTF and CAM. At 48 hr post-pFUS, only few CCTF, predominately anti-inflammatory in nature, remained elevated. By 72 hr post-pFUS, only M-CSF was elevated.

### pFUS does not stimulate homing of systemic macrophages to kidneys

**Figure 2:**
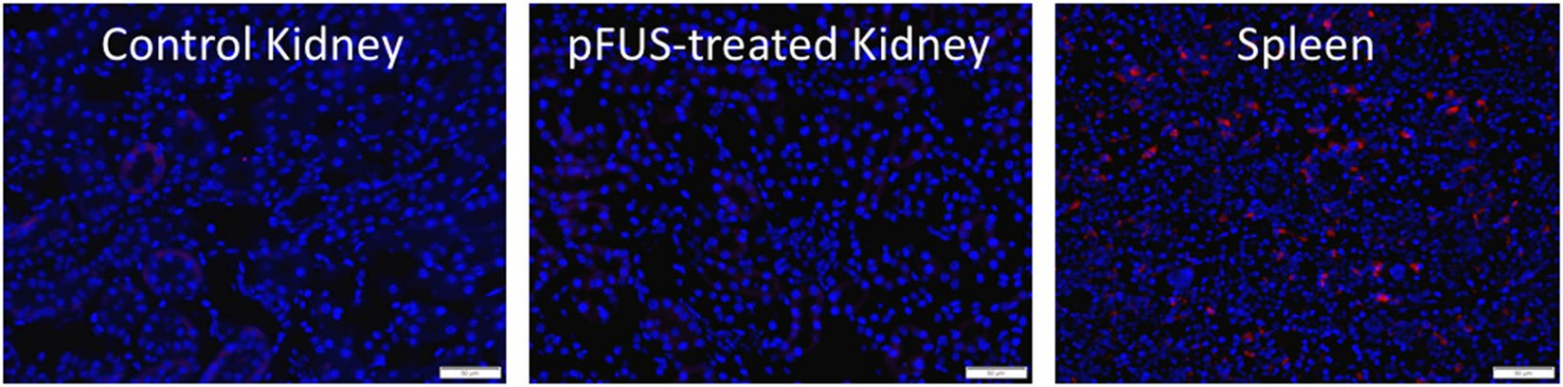
pFUS does not stimulate macrophage infiltration by 3 days post-pFUS. Mice were given fluorescently labeled SPION to label splenic macrophages 3 days before pFUS to kidneys. Mice were euthanized 3 days after pFUS. Treated and control kidneys were imaged and revealed rare incidences of SPION signal. For positive control, a spleen harvested 3 days post-pFUS demonstrates SPION fluorescence. Scale bars = 50 μm.

We investigated if systemic macrophages homed to pFUS-treated kidneys by pre-labeling splenic monocytes with rhodamine-labeled superparamagnetic iron oxide nanoparticles (SPION) 3 days before pFUS. At 3 and 5 days post-pFUS, SPION fluorescence was rarely observed in renal tissue from pFUS-treated kidneys or untreated control kidneys (p > 0.05) (Fig. 2). For positive controls, spleens on each day exhibited robust SPION fluorescence.

### pFUS does not cause adverse renal effects acutely or at 30-day followup

**Figure 3:**
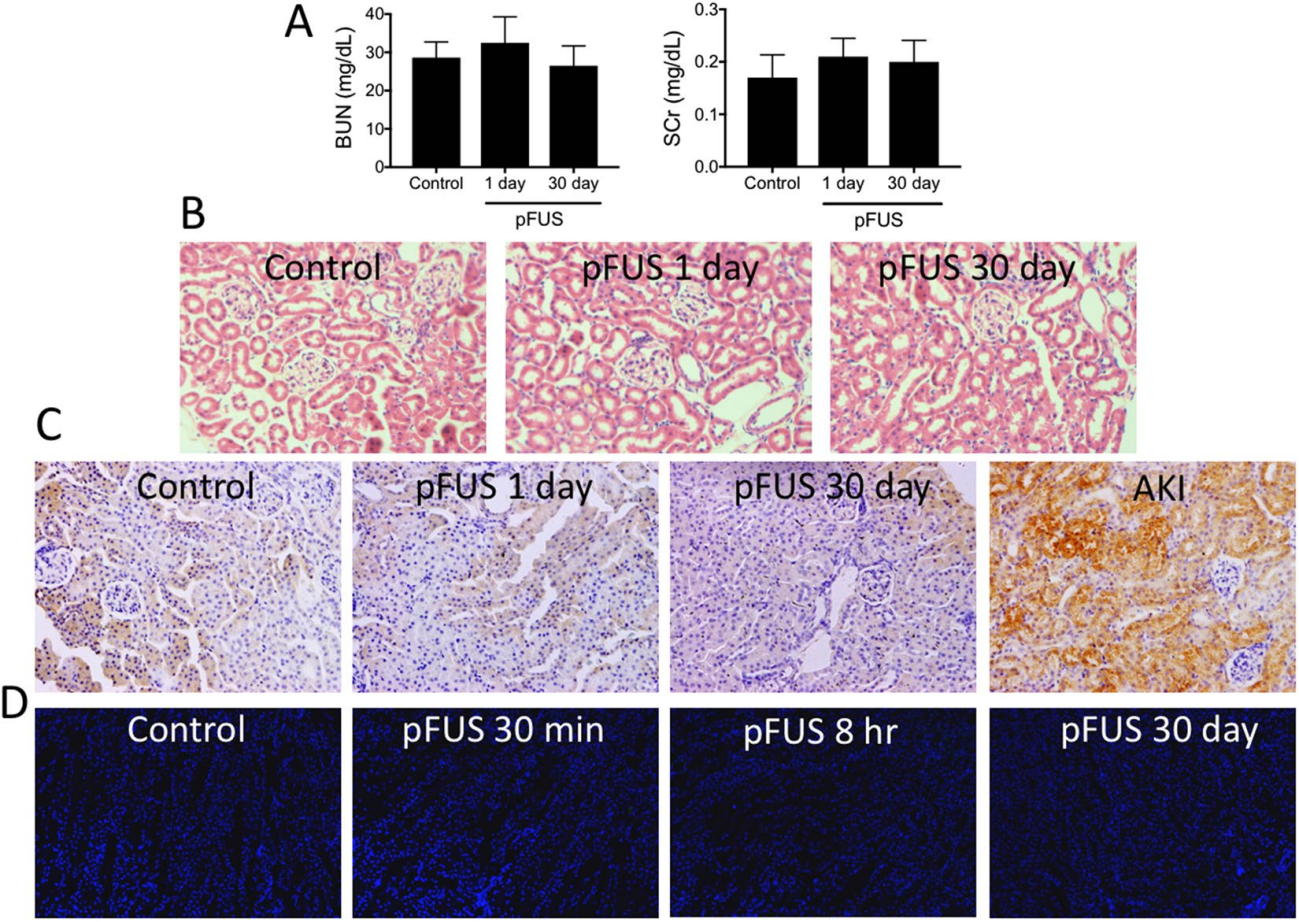
pFUS does not alter renal function, tissue architecture, or KIM1 expression at 1 or 30 days post-pFUS. (**A**) BUN and SCr values do not change between control mice (no pFUS) and mice at 1 or 30 days post-pFUS (p > 0.05; ANOVA). (**B**) H&E staining reveals consistent renal architecture between untreated controls and treated mice at 1 or 30 days. (**C**) Immunostaining for KIM1 demonstrates consistent expression between untreated controls and pFUS-treated kidneys at 1 or 30 days. For AKI positive control, kidneys were harvested 4 days after i.p. injection with cisplatin (15 mg/kg). The AKI kidney did not receive pFUS. (**D**) The appearance of TUNEL-positive cells in pFUS treated kidneys at 30 min, 8 hr, or 30 days post-pFUS was similar to unsonicated kidneys. Scale bars = 50 μm.

We previously demonstrated no adverse effects to pFUS in normal kidneys at 1 day post-sonication. Here, we investigated long-term effects of pFUS to 30 days post-sonication. Blood urea nitrogen (BUN) serum creatinine (SCr) levels were normal in mice 30 days after bilateral pFUS (Fig. 3a). Furthermore, hematoxylin and eosin (H&E) staining revealed no histological abnormalities at 30 days (Fig. 3b). Expression of kidney injury molecule-1 (KIM1) was found to be similar to untreated controls at 1 and 30 days post-pFUS (Fig. 3c). Both time points following pFUS also expressed less KIM1 than AKI kidneys 4 days after i.p. cisplatin injection (15 mg/kg), which served as positive controls. We found that pFUS to the kidney did not generate TUNEL-positive cells at 30 min, 8 hr, or 30 days post-sonication (Fig. 3d).

### Pretreatment with anti-inflammatory drugs suppresses renal molecular response to pFUS

**Figure 4:**
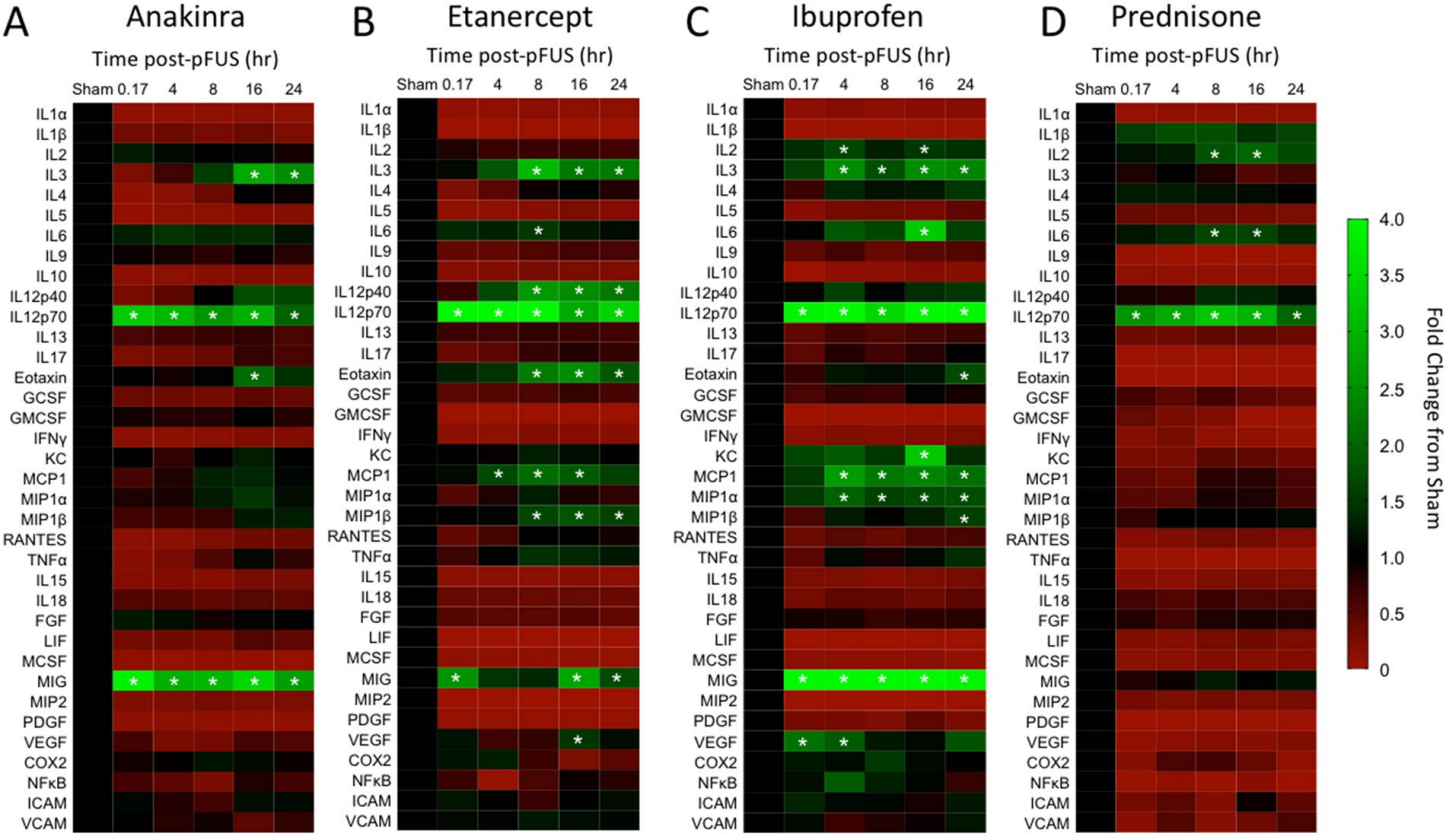
Anti-inflammatory drugs inhibit the molecular response to pFUS in kidneys. Heat maps showing molecular fold changes following pFUS when mice were pretreated with the indicated drug. Increases are indicated in green while decreases are shown in red. For each molecule, all time points were compared to a sham kidney (no drug, no pFUS) using ANOVA with Bonferroni post-hoc tests. Time points with p values < 0.05 were considered statistically significant and indicated by asterisks.

Since pFUS stimulated IL-1α and TNFα early, we postulated they induced the later molecular response. Therefore, we pharmacologically inhibited the molecular effects of each with FDA-approved drugs anakinra (IL-1α receptor antagonist) and etanercept (TNFα inhibitor) and measured the proteomic response in the presence of each drug at 10 min, 4, 8, 16, and 24 hr (n = 6 mice per time point per drug; total 144 mice). Cytokine levels at each time point were compared to sham-treated controls by ANOVA. Each drug was able to suppress large portions of the temporal pFUS molecular response (heat maps shown in Fig. 4a and b; see Supplement for primary data). Following pFUS in the presence of etanercept, VEGF was elevated at 16 hr, IL-6 was elevated at 8 hr, IL12-p70 was elevated at all time points, MCP-1 was elevated at 4, 8, and 16 hr, and IL3, IL12p40, eotaxin and MIP-1β were elevated at 8, 16, and 24 hr. Monokine induced by gamma interferon (MIG) was elevated at 10 min, and then again at 16 and 24 hr. Following pFUS in the presence of anakinra, IL-3 was elevated at 16 and 24 hr, and eotaxin was elevated at 16 hr. IL12p70 and MIG were elevated at all time points.

In pFUS-treated kidneys we also observed early upregulations of NFκB and COX2 following the initial increases of IL-1α and TNFα (Fig. 1). Therefore, we investigated the involvement of COX2, which is frequently stimulated by IL-1α and TNFα signaling via NFκB pathways. We surveyed the temporal proteomic response to pFUS in the kidney when mice were pretreated with ibuprofen (a non-selective COX inhibitor) or prednisone (a glucocorticoid that inhibits translocation of NFκB into the nucleus). Both of these drugs also inhibited large portions of the pFUS molecular response (heat maps shown in Fig. 4c and d). When ibuprofen was administered before pFUS, IL2 was elevated at 4 and 16 hr, IL3, MIP-1α, and MCP-1 were elevated at 4, 8, 16, and 24 hr, IL6 and KC were elevated at 16 hr, IL-12p70 and MIG were elevated at all time points, eotaxin and MIP-1β elevated at 24 hr, and VEGF was elevated at 10 min and 4 hr. In the presence of prednisone, IL-2 and IL-6 were elevated at 8 and 16 hr, and IL12p70 was elevated at all time points (Fig. 4).

### pFUS-induced tropism of MSC to kidneys is suppressed by pretreatment with anti-inflammatory drugs and in COX2-KO mice

**Figure 5:**
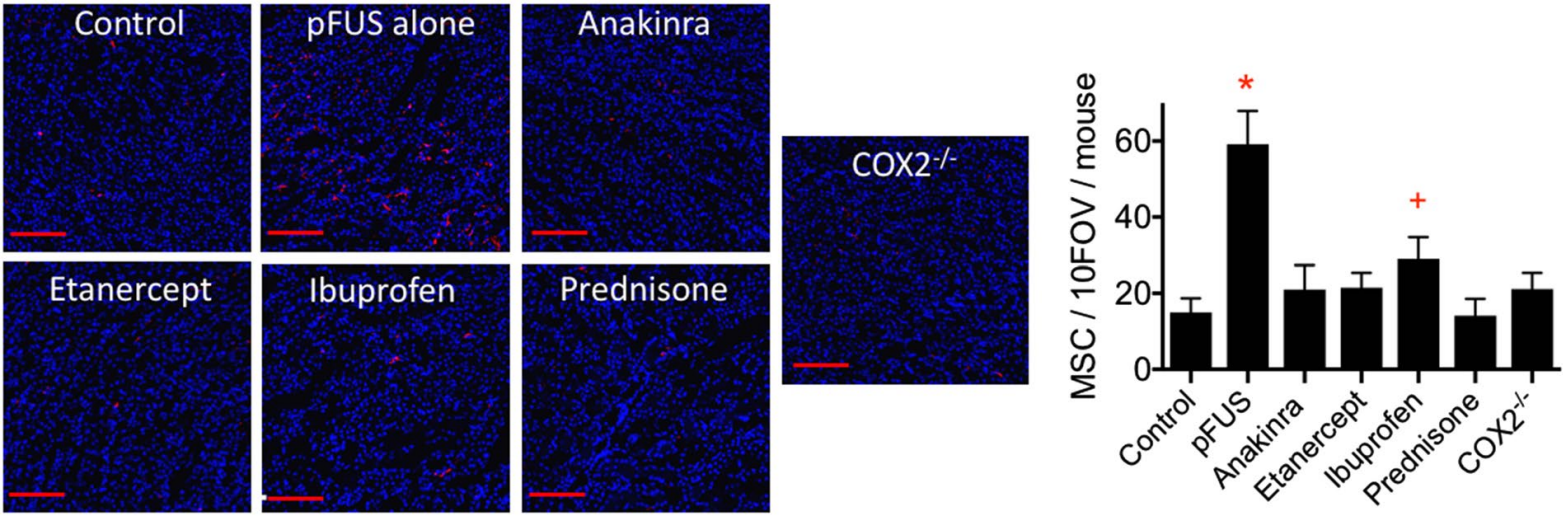
Renal tropism of MSC is suppressed by anti-inflammatory drugs and in COX2-KO mice. MSC do not routinely home to control kidneys (no pFUS, no drugs). Homing is enhanced ~5 fold in the pFUS-treated group (pFUS, no drugs). When anti-inflammatory drugs are administered prior to pFUS or pFUS is administered to COX2-KO mice without drug pretreatments, MSC tropism is suppressed compared to the pFUS alone group (p < 0.05). MSC homing to pFUS-treated kidneys of mice given ibuprofen was significantly greater than control kidneys (p < 0.05) but also significantly less than kidneys treated with pFUS alone. (n = 6 mice per treatment group; multiple comparisons made by ANOVA with Bonferroni post-hoc tests; symbols indicate statistically significant differences from groups with different or no symbol; Scale bars = 100 μm).

Kidneys in wild-type mice were unilaterally administered pFUS with or without anti-inflammatory drugs (n = 6 per group) and COX2-KO mice (n = 3) were given unilateral kidney pFUS alone in the absence of drugs. Four hours after pFUS, all mice were given i.v. infusion of 10^6^ human MSC. At 24 hr post-pFUS, treated and untreated kidneys were harvested and human MSC were quantified in kidneys by immunohistochemistry (IHC) for human mitochondria (Fig. 5). pFUS-treated ipsilateral kidneys were compared to untreated contralateral kidneys. In wild-type mice that received pFUS only (no drugs), MSC tropism was significantly increased to pFUS-treated kidneys (p < 0.05). Approximately 5 times more MSC were observed in pFUS-treated kidneys compared to contralateral control. These results were similar to previous studies reported by our group. When anti-inflammatory drugs were administered prior to pFUS and MSC, MSC tropism to pFUS-treated kidneys was suppressed. The numbers of MSC in pFUS-treated kidneys from animals that were given etanercept, anakinra, or prednisone were not significantly different (p > 0.05) from the numbers of MSC in control kidneys. The number of MSC observed in pFUS-treated kidneys following ibuprofen treatment was almost 2 times greater than in control kidneys (p < 0.05) but still significantly fewer MSC were observed compared to pFUS-treated kidneys of mice that did not receive drugs (p < 0.05). Furthermore, similar numbers of MSC were observed in pFUS-treated and control kidneys of COX2-KO mice (p > 0.05), which did not receive drugs.

## Discussion

**Figure 6:**
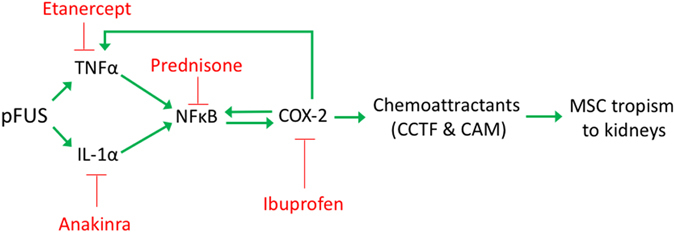
Schematic of pFUS-induced molecular response. Stimulation in the pathway is shown by a green arrows and inhibition by a red “T”. Blocking any particular point with a drug blunts the expression of chemoattractants and MSC tropism.

The primary findings of this study are that pFUS elicited a complex transient molecular response that increases tropism of MSC to kidneys that could be manipulated by FDA-approved anti-inflammatory drugs (Fig. 6). The evolution and propagation of the response required initial elevations of IL-1a, TNFa, to drive NFκB, and COX2 pathways. The molecular response induced tropism and EHPR of i.v.-infused MSC without mobilizing a systemic macrophage response, altering renal function or histological architecture, or increased KIM1 expression. Pretreating mice with the anti-inflammatory drugs anakinra, etanercept, prednisone, or ibuprofen, largely inhibited the renal molecular response to pFUS and suppressed MSC tropism to and EHPR in pFUS-treated kidneys. Lastly, MSC homing to kidneys was not increased by pFUS in COX2-KO mice, which were employed to specifically investigate the role of COX2 in MSC tropism since ibuprofen does not selectively inhibit COX2.

Ultrasound is being widely investigated as a therapeutic modality in renal regenerative medicine^12–16^. Many studies use microbubble contrast agents as cavitation nuclei and demonstrate enhanced tropism of stem cells to improve renal pathologies, but potential tissue damage from cavitation has not been adequately investigated. Diagnostic ultrasound has been shown capable of modulating neuroimmune axes to improve AKI^12^.

Although preclinical results are promising, little is known about the molecular biological responses following renal sonications. It is critical to understand the renal microenvironment given its importance for MSC tropism and *in vivo* mechanisms of action. We showed previously pFUS does not significantly raise temperature, but rather acoustic radiation forces and their mechanotransductive effects induce transient molecular responses across different tissues. We have previously characterized responses to similar sonications in muscle and show a fundamentally different response in the kidney. While the muscle response was biphasic, the renal response is monophasic. We show here that within 10 min, pFUS increases TNFα, IL-1α, and IL18 that could be considered a mild sterile inflammatory response. Shortly after, NFkB and COX2 pathways are activated and likely occur through well-defined pathways. Increased IL-18 is potentially due to its activation by caspase-1^17^ and could also have involvement in the molecular response. Early elevations in pro-inflammatory cytokines may result from mild cell stress from mechanical ultrasound forces that release alarmins, stressorins, or damage associated molecular patterns (DAMPS). It is also possible that mild stress is generated by brief hypoxia following sonications, which occurs in the brain by using microbubbles^18^. Future studies will investigate pFUS as a stressor, but it does not alter renal function, renal tissue architecture, or expression of KIM1, suggesting that while complex and dynamic, the molecular response is not damaging the kidneys. This is further supported in by our previous studies in AKI, where pFUS alone did not negatively impact the disease course of disease.

The initial molecular response following pFUS leads to increased expression of CAM from 4–24 hours. Four hours post-pFUS is the time of MSC infusions in this study. Cell adhesion molecules expressed on endothelial cells are generally necessary for tethering and subsequent extravasation of circulating MSC in response to CCTF gradients in the parenchyma. Between 8 and 16 hours post-sonication, the nature of the microenvironment switches from a generally pro-inflammatory to an anti-inflammatory profile. While the expression of COX2, TNFα, IL-1α, and NFkB returns to baseline, we detected increases in VEGF, PDGF, FGF, EPO, IL-10, and IL-15. This transition of the renal microenvironment from 8–72 hr post-sonication to increased expression of anti-inflammatory CCTF may be in response to the initial increases in TNFα, IL-1α, and IL18 but further investigation is needed. Furthermore, there may be subtle interactions between pro- and anti-inflammatory molecules should be investigated more thoroughly. For example, we demonstrate simultaneous elevations in EPO and IL-3 in the kidney. EPO has anti-inflammatory effects and is renoprotective, but its function is modulated by IL-3, which has been shown to suppress EPO-dependent red blood cell formation in anemic mice, but also work synergistically with EPO to increase survival in irradiated mice.

While pFUS mechanical effects induced a complex and dynamic molecular response in kidneys, it did not mobilize infiltration of systemic macrophages by 3 days post-sonication. This is different than what was previously measured for similar sonications in skeletal muscle using an identical macrophage labeling technique^6^. While there are similarities in the molecular responses between kidney and skeletal muscle, unique differences may account for the different macrophage tropism to targeted tissues. Moreover, following pFUS to muscle we observed a predominately anti-inflammatory (M2) macrophage phenotype in the tissue suggesting an influence of the induced CCTF on this cell population. Although, we have not been able to detect elevations in CCTF in the serum following pFUS to kidneys (data not shown), negative serum results could simply be from dilution effects. It is possible the CCTF and CAM response remains confined to the kidney and therefore, circulating immune cells would only be attracted to sonicated regions as they pass through the kidney. Further research is needed to investigate the cell type(s) (i.e., endothelial cells, pericytes, tubular cells, or resident immune cells) that are stimulated by pFUS to produces the various CCTF detected in the kidney.

In an effort to elucidate critical components of the pFUS-induced molecular response, we pretreated mice with pharmacological inhibitors etanercept, anakinra, prednisone, and ibuprofen. We found each drug administered before pFUS effectively shut down the proteomic response over 24 hours. Furthermore, blocking the molecular response with any of the drugs essentially suppressed MSC tropism to pFUS-treated kidneys. The lone exception was ibuprofen pretreatment. It did suppress tropism compared to pFUS-treated mice that did not receive drugs, but its effect was significantly less than the other drugs. In addition to being a nonspecific inhibitor of COX2, its effects are relatively weak and short-lived. Based on these results, we selectively probed COX2 by examining MSC tropism in COX2-KO mice and found that pFUS did not increase MSC tropism to targeted kidneys. It is important to note that pFUS to the kidneys followed by MSC infusion also resulted in suppression of the CCTF response in kidneys at 6 and 24 hours^11^. This finding would indicate that the mechanotransductive effects of pFUS to the kidney can be manipulated by both pharmacological and cellular therapeutics.

The drug effects on the pFUS molecular response reveal that early increased expressions of pro-inflammatory factors are critical to the later response, which is presumably the direct mediator of MSC extravasation. This study did not investigate which specific elements directly mediate MSC diapedesis, but does show that anti-inflammatory drugs inhibit MSC tropism to kidneys. These results may also be important in a wider context of renal MSC therapies. MSC tropism during disease still depends on microenvironment of diseased kidneys, which may share similarities with the post-pFUS kdiney. Therefore, concurrent administration of MSC and drugs may have similar effects on MSC tropism during the pFUS response. Further empirical investigation of drugs and renal diseases is necessary. This is especially important in kidney transplantation, where MSC are being widely investigated to minimize transplant rejection or graft-versus-host disease (GVHD). Most clinical MSC trials do not control for drugs, which could be sources of variation in clinical effectiveness.

While this study examined organ/tissue-level effects of pFUS and drugs, future studies will need to examine the cellular components to the pFUS-induced molecular response. This response presumably represents contributions made from renal tubular epithelium and fibroblasts, renal endothelium, and infiltrating/resident immune cells. For example, the early part of the molecular response has substantial overlap with the well-characterized NLRP3 inflammasome that is well characterized in the kidney^19^. IL18, IL1, and NFκB are all critical elements of this response and observed immediately after pFUS. NLRP3 responses are well documented to occur initially in renal dendritic cells^20^ and to a lesser extent, tubular^21^, and glomerular cells^22^. Cell adhesion molecules are presented and upregulated by endothelial cells^11^ and they presumably contribute proangiogenic factors like VEGF, which is also produced by podocytes^23^. Many of the growth factors, chemokines, and anti-inflammatory cytokines we observed in the later part of the response could be due to infiltrating regulatory T-cells (T_reg_). While these factors are known to be secreted by macrophages with M2 phenotypes^24^, we did not observe macrophage infiltration in kidneys. T_reg_ cells were not investigated in this study, but they are known to secrete many similar factors as M2 macrophages^25^ and are currently under investigation by us.

In conclusion, this study characterizes the molecular response from pFUS in kidneys that can be capitalized to increase MSC tropism. Early elevations in TNFα, and IL-1α drive the molecular response presumably through NFkB- and COX2-dependent pathways although other molecular mechanisms need to be explored. Pretreating mice with anti-inflammatory drugs interferes with the evolution of the response and ultimately suppresses homing of i.v.-infused MSC. This study demonstrates that the renal microenvironment is critical to MSC tropism and could have profound impacts on MSC therapies. Common drugs are shown to interfere with this process and their effects will need to be understood as ultrasound technologies become further utilized in regenerative medicine, but also how they impact stem cell therapies where renal tropism is important for therapeutic efficacy.

## Materials And Methods

### Animals

All animal studies were approved by the animal care and use committee at our institution, and experiments were performed according to the National Research Council’s Guide for the Care and Use of Laboratory^26^. Female C3H mice (Charles River Laboratories, Wilmington, MA) and B6;129S-*Ptgs2* ^*tm1Jed*^/J (COX-2^−/−^ mice; Jackson Laboratory, Bar Harbor, ME) were aged 10–14 weeks and allowed free access to food and water during studies.

### Pulsed Focused Ultrasound (pFUS)

pFUS was performed with a VIFU 2000 (Alpinion Medical Systems, Bothell, WA) using ultrasound imaging guidance while mice were anesthetized with 2.5% isoflurane in 100% O_2_ via nosecone. Unilateral kidney pFUS was administered with the following parameters: peak negative pressure = ~4.0 MPa; pulse repetition frequency, 5 Hz; duty cycle, 5%; number of pulses per site, 100.

### Drugs and SPION

Prior to pFUS exposures, mice were treated with one of the following drug regimens: Ibuprofen (Perrigo, Allegai, MI) at 30 mg/kg p.o. 15 min before pFUS; Etanercept (Amgen, Thousand Oaks, CA) at 4 mg/kg i.p. 3 days and 1 day before pFUS; Anakinra (Amgen) at 100 mg/kg i.p. 2 days, 1 day, and 1 hr before pFUS; Prednisone (Hi Tech Pharmacal, Amityville, NY) at 10 mg/kg p.o. 2 days, 1 day, and 2 hr before pFUS. Some mice had AKI induced by i.p. injection of cisplatin (15 mg/kg) and were euthanized 72 h post-injection. Molday ION Rhodamine-B-labeled SPION (Biopal, Worcester, MA) were injected i.v. at 8 mg Fe/kg body weight 3 days before pFUS.

### MSC cultures and injections

Human MSC were cultured in α-minimum essential medium supplemented with fetal bovine serum (20%). MSC were trypsinized and resuspended at a concentration of 10^7^ cells/mL in Hank’s balanced salt solution (HBSS) containing 10 U/mL sodium heparin. Approximately 4 hr post-pFUS, mice were given an intravenous (i.v.) injection of sodium nitroprusside (1 mg/kg) followed immediately by i.v. infusion of 10^6^ human MSC.

### Tissue harvesting and sample preparation

For molecular analyses, mice were euthanized and kidneys were immediately frozen in liquid N_2_. Tissue was homogenized in cell lysis buffer (Cell Signaling Technology, Danvers, MA) containing protease inhibitors (Santa Cruz) using a Bead Beater (BioSpec, Bartlesville, OK). Homogenized samples were centrifuged for 30 min at 4 °C and the supernatants were frozen at −80 °C for later use. Total protein concentrations were determined using bicinchoninic acid assays. For histological sample collection, mice were perfused with ice-cold PBS containing 4% (w/v) paraformaldehyde (PFA). Kidneys were harvested and stored in excess PFA for 24 hr at 4 °C and then transferred to PBS. Tissues were embedded in paraffin and sectioned at 3 μm thickness.

### Histological Analyses

Antigen retrieval was performed with 10 mM citrate buffer (pH 6.0) and sections were blocked with SuperBlock (ThermoFisher Scientific, Waltham, MA). Primary antibodies against human mitochondria (1:200) or murine KIM1 (1:500) (Abcam) were used with species-appropriate, horseradish peroxidase (HRP)- or Alexafluor 647-labeled secondary antibodies. HRP-labeled antibodies were exposed to 3,3’-diaminobenzidine (DAB) for 2 minutes. Hematoxylin and eosin staining was performed according to standard protocols. TUNEL was assayed using an *In Situ* Cell Death Detection Kit—Fluorescein (Sigma Aldrich) according to manufacturer protocols. Histological sections requiring quantitative analyses of features (human mitochondria and TUNEL) were counted across 10 high-power fields-of-view (FOV) from 3 slides per animal. Evaluators were blinded to the experimental conditions of each section.

### Microscopy

Slides were viewed with an Aperio FL Slide Scanner (Leica Biosystems, Nussloch, GmbH) with appropriate excitation and emission filters or Olympus BX61 light microscope (Olympus, Shinjuku, Japan).

### Molecular Analyses

Cytokines were measured in kidney tissue using a Bio-Plex multi-plexed enzyme-linked immunosorbant assay (ELISA) (Mouse Cytokine 23-plex and Mouse Cytokine 9-plex; Bio-Rad, Hercules, CA). Single-plexed ELISAs for COX2, ICAM, VCAM, and NFκB (Raybiotech, Norcross, GA), and COX2 (R&D Systems, Minneapolis, MN) were also performed. ELISAs were loaded with tissue homogenates at a concentration of 2 mg/mL and developed according to manufacturer protocols.

### Renal Function Measurements

Renal function was measured using assay kits (Sigma Aldrich, St. Louis, MO). Blood urea nitrogen (BUN) was measured spectrophotometrically following degradation by urease. Serum creatinine (SCr) was measured fluorometrically following degradation by creatininase; this assay reports results similar to high-pressure liquid chromatography methods.

### Statistical Analyses

Data are presented as the mean ± standard deviation. Multiple comparisons were made using one-way analysis of variance (ANOVA) with Bonferroni post-hoc tests. All statistical tests were two-sided and p values < 0.05 were considered significant.

## Electronic supplementary material

Supplemental Data
