# Supplementary material for: Anti-inflammatory drugs suppress ultrasound-mediated mesenchymal stromal cell tropism to kidneys
Source: Sci Rep. 2017 Aug 17;7:8607. doi: 10.1038/s41598-017-08887-x (PMC5561076; doi:10.1038/s41598-017-08887-x)
Supplement: Supplementary file 1 — Supplemental Data [file 41598_2017_8887_MOESM1_ESM.pdf]

SUPPLEMENTAL DATA AND METHODS FOR:

Anti-inflammatory drugs suppress ultrasound-mediated mesenchymal stromal cell tropism to kidneys

Scott R. Burks[1], Ben A. Nguyen[1], Michele N. Bresler[1], Matthew E. Nagle[1], Saejeong J. Kim[1], and Joseph A. Frank[1,2]

[1]Frank Laboratory, Department of Radiology and Imaging Sciences, National Institutes of Health Clinical Center, Bethesda, MD 20892

[2]National Institute of Biomedical Imaging and Bioengineering, Bethesda, MD 20892

Address Correspondence to:

Scott R. Burks, Ph.D.

10 Center Dr. RmB1N256

Bethesda, MD 20895

scott.burks@nih.gov

phone (301) 594-2368

fax (301) 594-3216

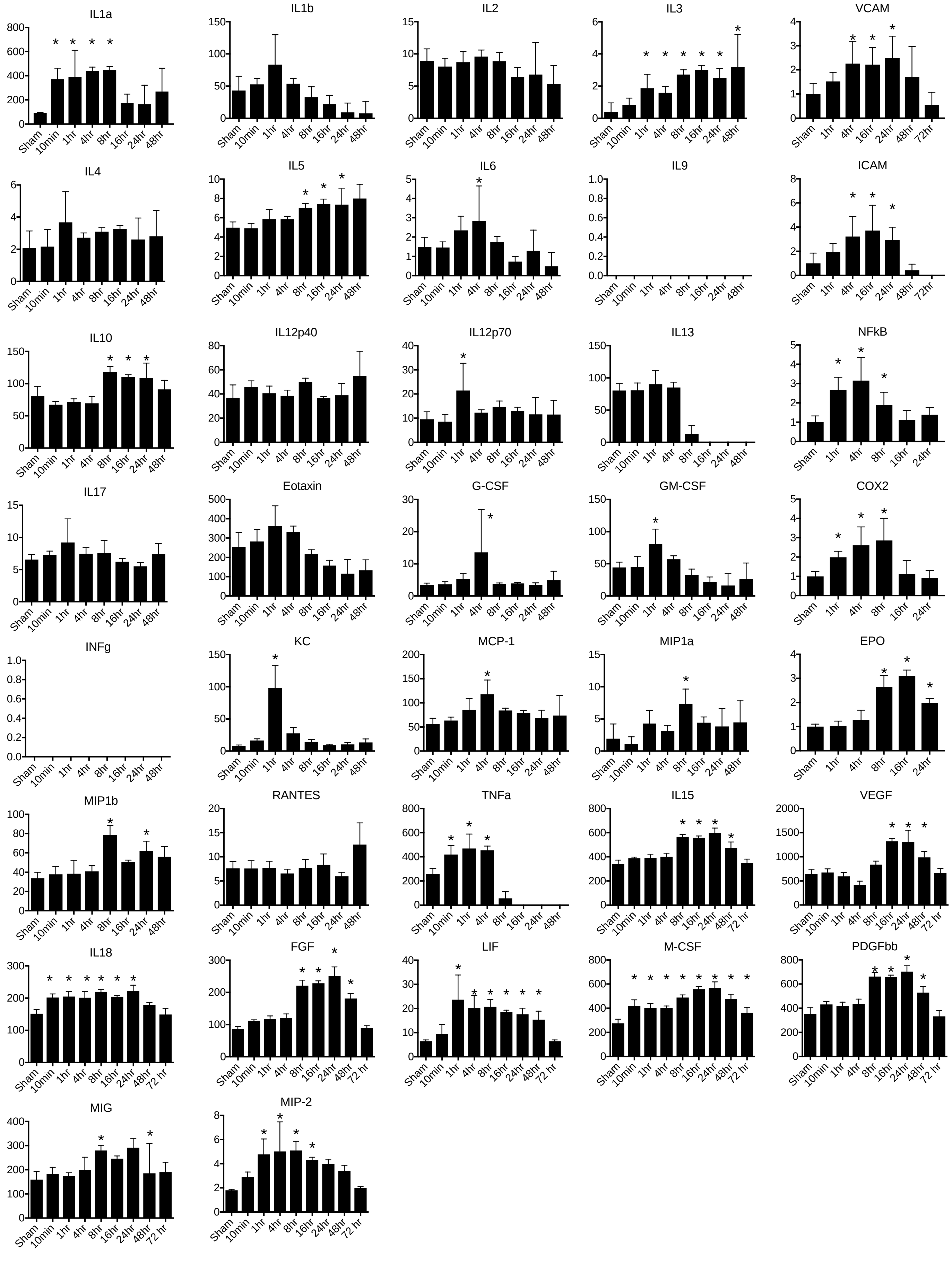

Supplemental Figure 1. Temporal changes in cytokines chemokines, trophic factors, and cell adhesion molecules following pFUS to the kidney. Y axes indicate concentration in pg/mL. Asterisks indicate statistically significant increases (p <0.05) from sham (no pFUS) controls by ANOVA using Bonferonni post-hoc analyses.

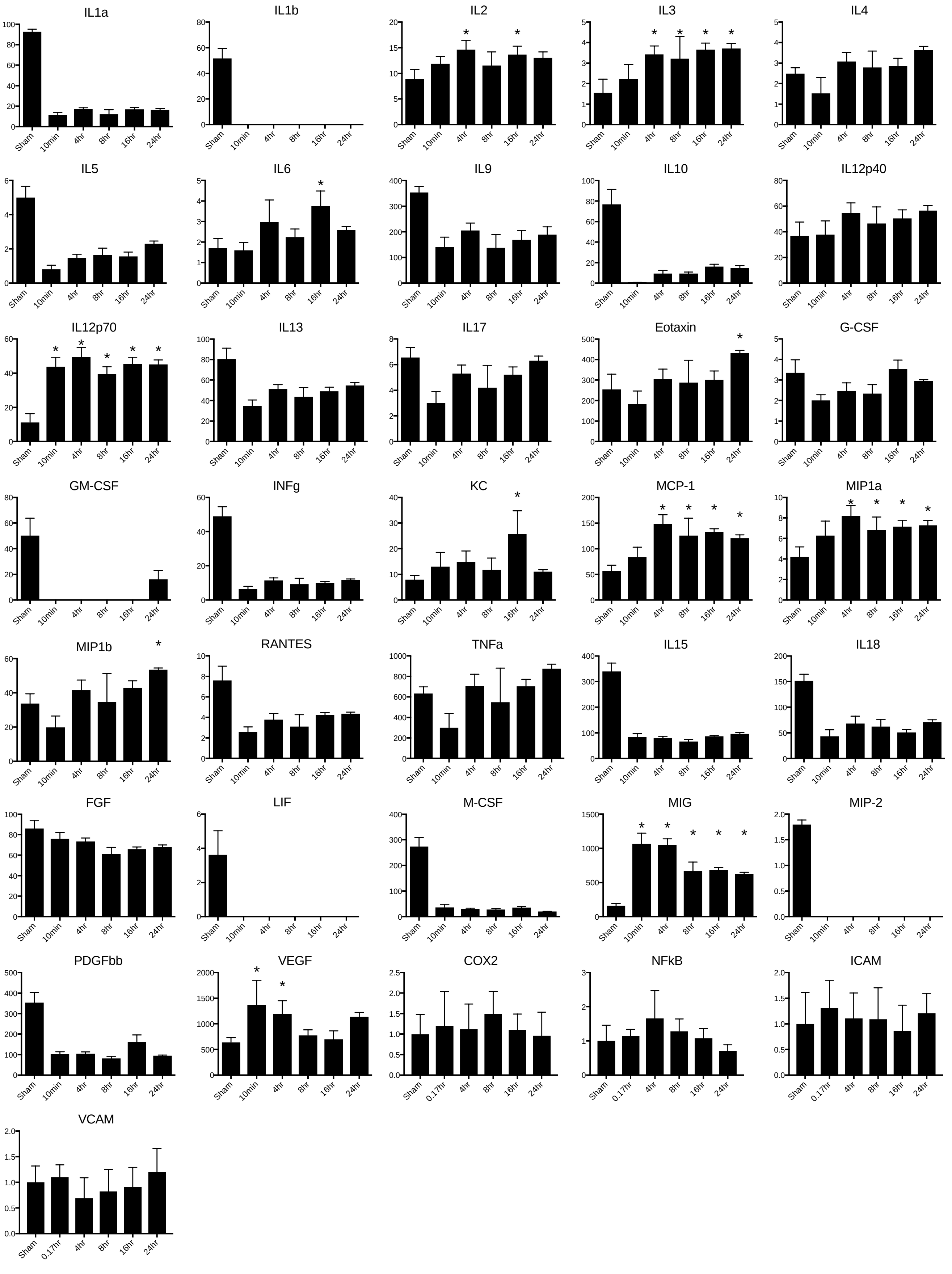

Supplemental Figure 2. Temporal changes in cytokines chemokines, trophic factors, and cell adhesion molecules following pFUS to the kidney when mice were pretreated with ibuprofen (see methods for details). Y axes indicate concentration in pg/mL. Asterisks indicate statistically significant increases ($p < 0.05$) from sham (no drugs, no pFUS) controls by ANOVA using Bonferonni post-hoc analyses.

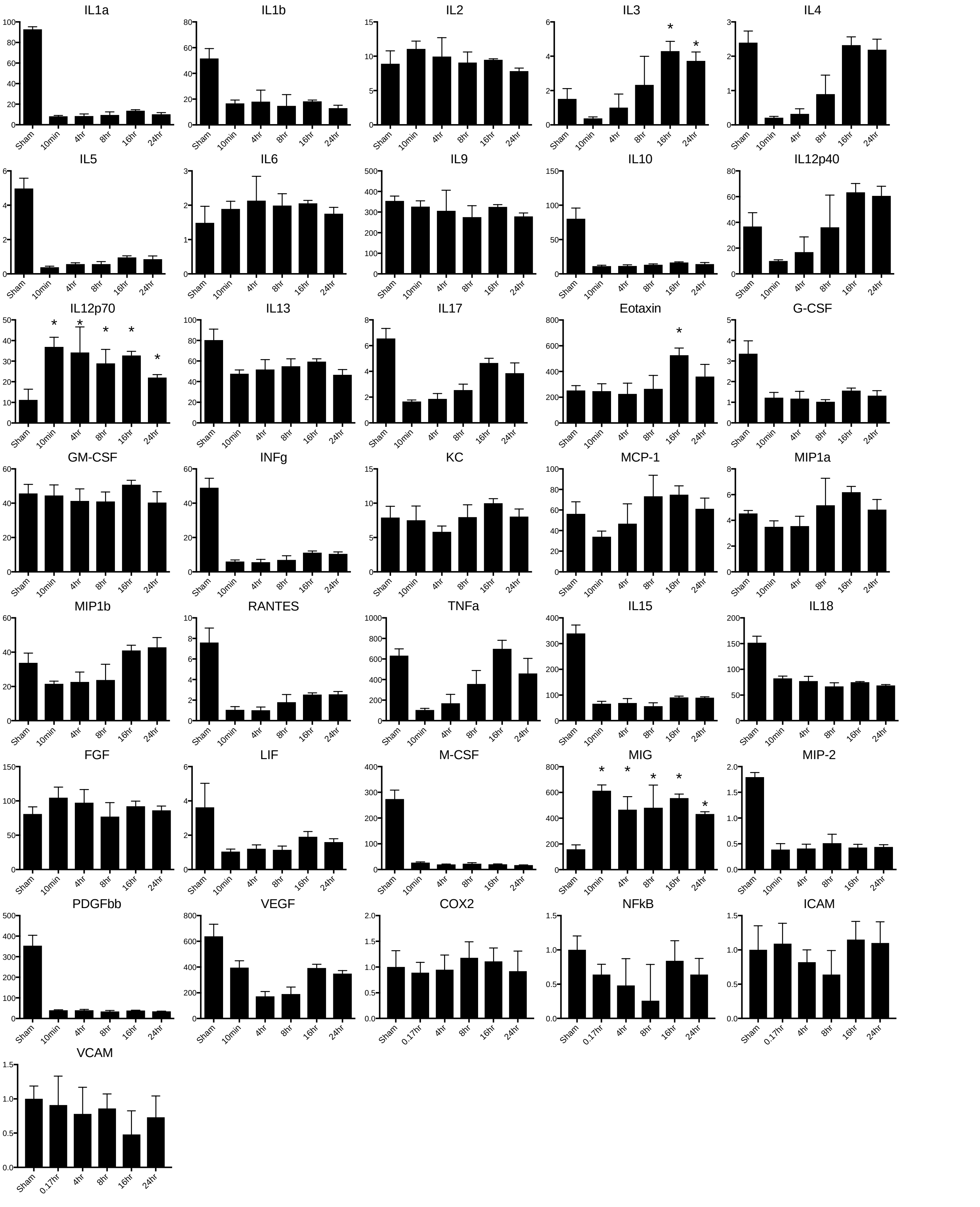

Supplemental Figure 3. Temporal changes in cytokines chemokines, trophic factors, and cell adhesion molecules following pFUS to the kidney when mice were pretreated with anakinra (see methods for details). Y axes indicate concentration in pg/mL. Asterisks indicate statistically significant increases ($p < 0.05$) from sham (no drugs, no pFUS) controls by ANOVA using Bonferonni post-hoc analyses.

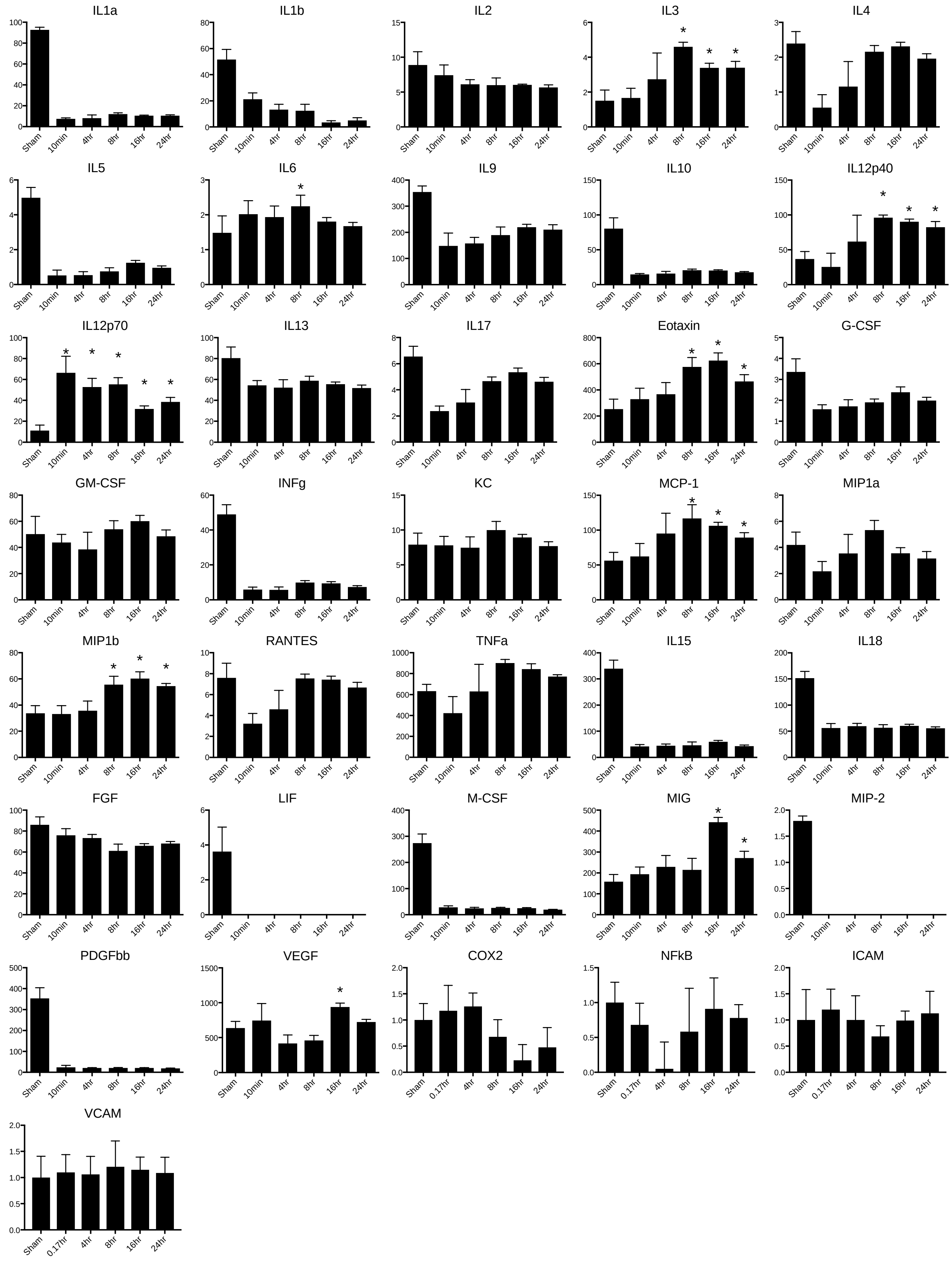

Supplemental Figure 4. Temporal changes in cytokines chemokines, trophic factors, and cell adhesion molecules following pFUS to the kidney when mice were pretreated with etanercept (see methods for details). Y axes indicate concentration in pg/mL. Asterisks indicate statistically significant increases ($p < 0.05$) from sham (no drugs, no pFUS) controls by ANOVA using Bonferonni post-hoc analyses.

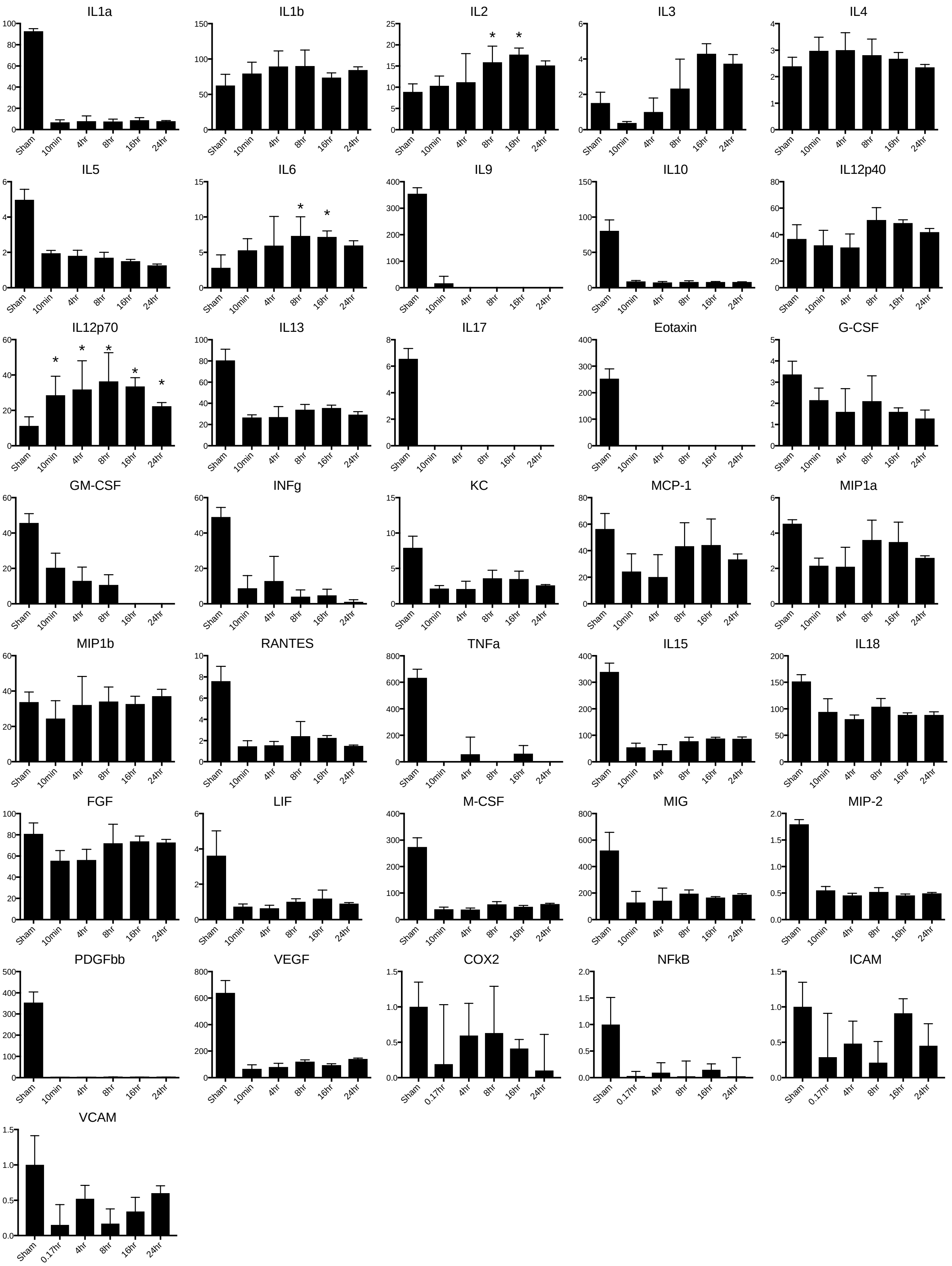

Supplemental Figure 5. Temporal changes in cytokines chemokines, trophic factors, and cell adhesion molecules following pFUS to the kidney when mice were pretreated with prednison (see methods for details). Y axes indicate concentration in pg/mL. Asterisks indicate statistically significant increases (p <0.05) from sham (no drugs, no pFUS) controls by ANOVA using Bonferonni post-hoc analyses.
